# Quantification of *Trypanosoma brucei* social motility indicates different colony growth phases

**DOI:** 10.1098/rsif.2024.0469

**Published:** 2024-12-18

**Authors:** Andreas Kuhn, Timothy Krüger, Magdalena Schüttler, Markus Engstler, Sabine C. Fischer

**Affiliations:** ^1^ Center for Computational and Theoretical Biology, Julius-Maximilians-Universität Würzburg, Biocenter, Klara-Oppenheimer-Weg 32, Würzburg 97074, Germany; ^2^ Department of Cell and Developmental Biology, Julius-Maximilians-Universität Würzburg, Biocenter, Am Hubland, Würzburg 97074, Germany

**Keywords:** social motility, *Trypanosoma brucei*, colony expansion, image analysis, emergent behaviour, ranged expansion

## Abstract

*In vitro* colonies of the flagellated parasite *Trypanosoma brucei* exhibit characteristic fingering instability patterns. To enable data-driven and data-validated mechanistic modelling of these complex growth processes, it is crucial to first establish appropriate quantitative metrics beyond qualitative image comparisons. We present a quantification approach based on two scale-free metrics designed to characterize the shape of two-dimensional colonies. Originally developed for yeast colonies, we adapted, modified and extended this analysis pipeline for the *Trypanosoma* system. By combining these quantitative measurements with colony growth simulations based on the Eden model, we identified two distinct growth phases in social motility-exhibiting colonies: an initial phase of mainly circular expansion, followed by a transition to an almost exclusive finger-growing phase. These phases remain robust with increasing cell numbers and upon partial inhibition of finger formation. A newly developed anisotropy index reveals that partial inhibition leads to increased colony anisotropy over time. Our results provide objective measurements that advance the understanding of social motility and serve as a foundation for future mechanistic modelling efforts. Furthermore, our approach offers a blueprint for investigations of other colony-forming microorganisms, such as yeast or bacteria, emphasizing the broader applicability of developing appropriate metrics for complex biological phenomena.

## 1. Introduction

Densely packed *Trypanosoma brucei* colonies on agarose gel surfaces exhibit a phenomenon known as social motility (SoMo) [1,2]. These colonies initially manifest as circular structures but upon expanding produce characteristic fingering instabilities. The fingers or protrusions grow in a distinct perpendicular alignment away from the colony’s centre.

SoMo in *T. brucei* shares similarities with bacterial swarming, a flagella-induced collective movement observed across various bacterial genera [3–6]. While environmental factors influence bacteria swarming, different species show very diverse expansion patterns. This suggests that swarming is closely tied to species-specific motility mechanisms and behaviours [5]. These parallel phenomena provide context for understanding *T. brucei*’s social motility while emphasizing the need for system-specific analyses.

Numerous studies have delved into various facets of SoMo in trypanosomes over the years. Imhof [7] demonstrated that SoMo is characteristic of the early procyclic form of *T. brucei*. Subsequently, a pivotal role of intracellular cAMP levels in regulating SoMo [8,9] has been proposed. Further, a correlation between the ability to collectively migrate *in vitro* and the development of trypanosomes in the tsetse fly has been identified [10]. As a potential method of communication between cells, necessary for the proposed social aspect of the collective motion, secreted exosomes have been shown to have a repulsive effect on active colonies [11]. Finally, an actual positive chemotaxis was proposed to be caused by bacterial colonies [12], and positive as well as negative pH-Taxis was demonstrated [13]. The first quantification of single-cell motility during collective migration, surprisingly, showed minimal correlation with the overall migration speed. These results highlighted the importance of as-yet-unclear physical parameters underlying the phenomena of SoMo [14].

Despite the noteworthy findings in these studies, a crucial aspect has been overlooked: a quantification of the investigated SoMo phenomenon at the level of the whole colony. Previous attempts have primarily relied on binary manual classification of SoMo-negative and SoMo-positive colonies. Various metrics have been explored, including cell number and density measurements [7,10], finger or protrusion counts [8–10] and changes in distance between interacting colonies [11], alongside a more sophisticated chemotactic index [12] and single-cell velocity measurements together with summary statistics [12,14]. However, most of these quantifications only capture specific facets of the SoMo phenomenon, and even the more intricate ones, like the chemotactic index, do not allow the quantification of the complete migratory behaviour of the system.

Therefore, we propose a quantification method solely based on colony morphology, independent of other measured variables and applicable across all demonstrated SoMo assays. This method builds on metrics introduced for yeast colonies [15] and assigns numerical values to colonies, indicating their degree of SoMo activity, rather than relying on categorical statements. To get a better understanding of the measurements, we introduced simulated colonies based on a modified version of the Eden model [16]. This combination of quantification and simulation of colony morphology enhances the comparability between experiments and allows for a more nuanced exploration of the influence of different parameters on SoMo dynamics.

Our pipeline is scale free, utilizing segmented image data and is openly available on GitHub and easily adaptable by other research groups. By providing a comprehensive, quantitative characterization of SoMo, our work establishes a foundation for future studies to explore the underlying mechanisms of this complex biological phenomenon in a more systematic and comparable manner.

## 2. Methods

**Figure 1.**
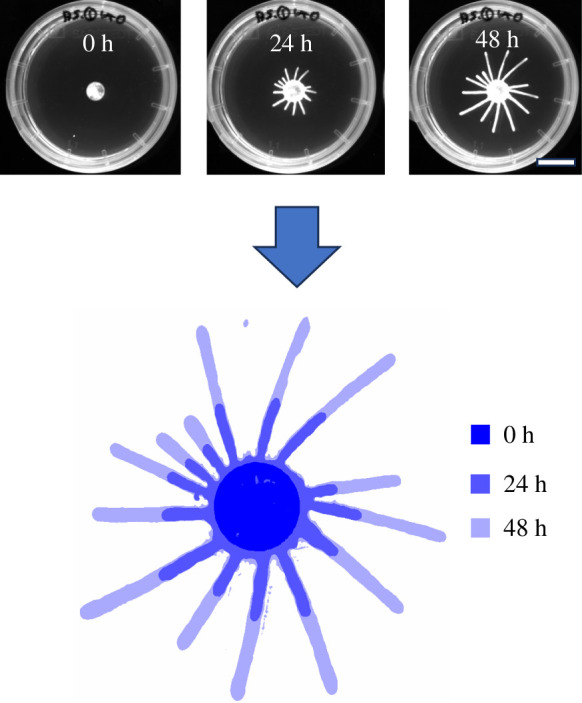
Image analysis pipeline transforms time-series fluorescence images (top, scale bar 20 mm) into segmented and aligned stacks of binary images (bottom) for further processing. At the bottom, time points are indicated by pseudocolouring.

We developed an image analysis pipeline to quantify the changes in colony morphology observed in fluorescent images (figure 1). To facilitate comprehension of the findings, we generated artificially grown colonies based on the Eden growth model [16] representing extreme scenarios across various growth regimes and compared their behaviour with that of real colonies using adapted metrics initially introduced by Binder [15] for the quantification of spatial growth patterns of yeast colonies.

### 2.1. Cell culture, motility assay and image acquisition

Cell culture and motility assays were performed essentially as in [14] with the following specifics. AnTat 1.1 and AnTat 1.1 tdTomato cells were grown in SDM-79-glc supplemented with 10% fetal bovine serum (FBS). Twenty per cent of fluorescent cells (expressing tdTomato) were added to the wild-type AnTat 1.1 cells before each experiment. The trypanosomes were concentrated to a density of 4 × 10^8^ cell ml^−1^ of which 2 µl per colony were dropped onto an agarose gel. This concentration was calculated to result in $10^{6}$ cells spread in a monolayer of $30\;mm^{2}$ surface area, concentrated at 1.7 × 10^10^ cell ml^−1^ in a colony drop volume of 0.06 µl after absorption of the culture medium by the hydrogel. For the experiment in figure 11, the initial concentration was five times the calculated monolayer amount and double the total amount of cells, resulting in faster initiation of projections. We made use of the inherent variability in motility and expansion behaviour of the pleomorphic cell line AnTat 1.1 to drop SoMo positive and SoMo negative colonies on the agarose gel. For gel production, 1 ml SDM-79-glc containing 0.4% agarose was poured in 35 mm Petri dishes, left to gel 1 h with a closed lid and dry for 30 min without lid under a laminar flow hood. Gels were recorded with the iBright CL1000 imager (Thermo Fisher Scientific).

### 2.2. Image analysis pipeline

Our primary objective in quantifying colony morphology change/growth is to analyse the alteration in occupied area over time. Hence, the image analysis pipeline aims to accurately segment colonies from the background and align individual colonies across the time series (figure 1).

Segmentation was performed using the machine learning-based Fiji plugin, Trainable Weka Segmentation [17,18] resulting in binary images where pixels with the value one belong to the colony and those with the value zero do not. Subsequently, alignment and stacking were executed using the Rigid Body transformation type of the Fiji plugin StackReg [19]. As alignment failure occurred in approximately 20% of images, a robust yet imprecise backup approach was implemented in the programming language Julia [20]. This approach utilizes convolutions to approximate the original centre of the colony at t=0 for all measured time-series images of a colony. For a detailed description of the image analysis pipeline, see appendix A.

### 2.3. Artificial colonies

In line with our focus on establishing appropriate metrics for data-driven mechanistic modelling, we sought to gain a deep understanding of our measured data and develop meaningful quantifications. To achieve this, we employed a modified version of the phenomenological Eden growth model [16] to generate artificial colonies. Our goal was to simulate three distinct growth modes and compare them with real colonies using our newly developed quantification methods (see §§2.4 and 3). This approach serves two crucial purposes. First, it enhances our understanding of the introduced quantifications by demonstrating how they behave in well-defined edge cases. Second, it provides a valuable context for interpreting real colony behaviour by allowing step-by-step comparisons between simulated and observed growth patterns.

#### 2.3.1. Eden model

The Eden model models the growth of clusters from predetermined seeds through the stochastic accumulation of particles on their surface. While originally formulated to mimic the growth of bacterial colonies [21–23], the Eden model has found applications in diverse biological systems, encompassing processes like tissue accumulation in wound healing [24] or the growth of acellular slime moulds [25]. Moreover, it extends its utility to the growth of clusters in pure physical systems, such as the formation of charged particles [26].

**Figure 2.**
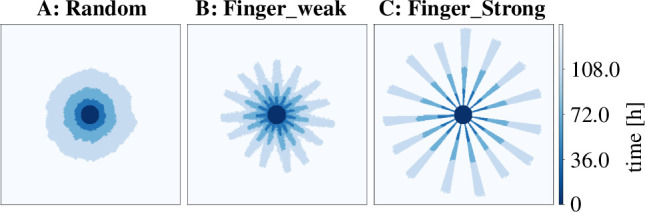
Examples of artificially created colonies. (*a*) The classical Eden model with solely random growth, (*b*) is a mixture of random growth and growth restricted to 15 directions and (*c*) allows growth only in 15 restricted circular sections. All models are normalized to the same amount of area growth as obtained from the previous section.

We used the area growth dynamics obtained in §3.2 and simulated three versions of the on-lattice Eden model. The initial version (version A) adheres to the classical Eden model, where in each growth step a growth site is randomly chosen that is empty and has to be neighbouring at least one site that is part of the colony. The second variant (version B) combines elements of the classical Eden model with partially directional growth. For a given average number of fingers, a corresponding number of circular sections with a similar width are designated as preferred growth directions with higher probability of site selection. Version C of the model builds upon this concept by restricting growth exclusively to these preferred directions. At the onset of each simulation, the directions of the circular sections are randomly generated and roughly evenly distributed across the colony surface (figure 2).

Since time resolution in these artificial colonies is only limited by computation time, we chose a much more fine-grained time resolution of 2 h increments as compared with the experimental data. We performed simulations for 128 colonies for each version. These simulations resulted in binary images that can be directly compared with the experimental images processed by the image analysis pipeline (figure 1). Details of the implementation can be found in appendix C.

### 2.4. Metrics

A binary colony image can be conceptualized as a matrix M(x,y) with


(2.1) $$\begin{array}{ccc} & M(x,y)=\left\{ \begin{array}{cc} 1, & \text{part of the colony},\\ 0, & \text{not part of the colony}, \end{array}\right. & \end{array}$$

where (x,y) represents the position of a pixel. Additionally, we define the set of vectors $V_{all}$, which encompasses all vectors $\vec{v}(x,y)$ pointing towards a pixel that is part of the colony. With these definitions, the centroid s of each colony can be computed as


(2.2) $$\begin{array}{r} \vec{s}(x_{s},y_{s})=\frac{1}{m}\sum_{i=0}^{m}{\vec{v}}_{all,i}(x,y) \end{array}.$$

Applying these definitions to the segmented binary images, we computed binary net growth images $M_{net}(t)$ that only consist of the additional area grown in a colony and their respective set of vectors $V_{net}$ relative to the centroid $\vec{s}$ at t=0. If not specifically mentioned otherwise, all vectors used in further calculations are always from the set $V_{net}$


$$M_{net}(t)=M(t)-M(0),$$

$$V_{net}=\{\vec{v}(x,y)|M_{net}(x+x_{s}(t=0),y+y_{s}(t=0))=1\}.$$

#### 2.4.1. Angular metric

**Figure 3.**
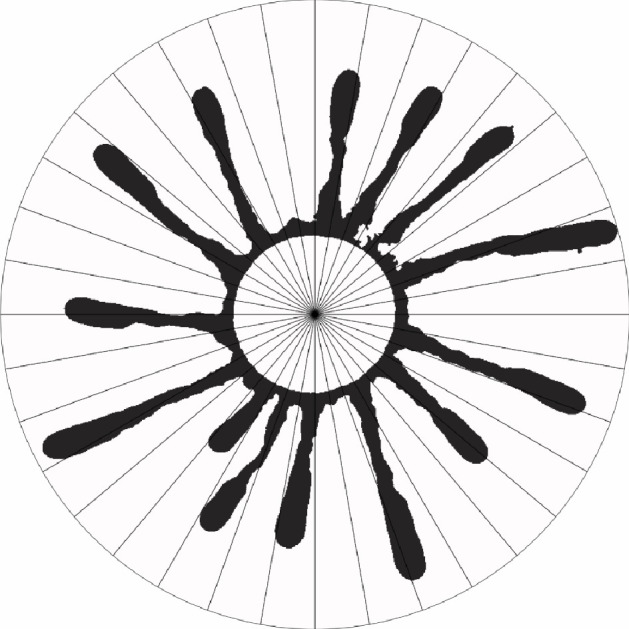
Example net growth image $M_{net}(48h)$ of a colony divided into 36 circular sectors (for illustration) originating from the centre of the original circular shaped colony at *T =* 0 h. To calculate the angular metric, the black pixels are counted in each sector.

**Figure 4.**
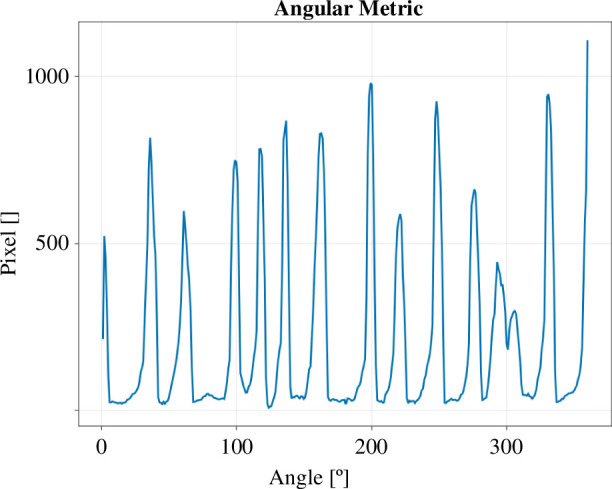
Angular metric for *n* = 360, for the net growth image $M_{net}$ of the colony shown in figure 3. Each of the peaks corresponds to a finger in the image.


The angular metric $S_{\theta }(i)$ describes the radial variations of a colony. It is calculated by dividing the net growth binary images $M_{net}$ into N circular sectors (figure 3) and counting the number of occupied pixels inside each sector (figure 4). The centroid $\vec{s}=\left(\begin{array}{c} x_{s}\\ y_{s} \end{array}\right)$ of the initial image of a colony serves as the centre of the circular sectors for all images in one time series. We get

(2.3) $$\begin{array}{c} S_{\theta }(i)=\{\vec{v}(x,y)|\\ \frac{2\pi }{N}i\leq tan^{-1}\left(\begin{array}{c} x\\ y \end{array}\right)<\frac{2\pi }{N}(i+1),\vec{v}\in V\}\\ \text{for i }=0,1,...,N-1 \end{array}.$$

In this work, we chose N=360.

#### 2.4.2. Pair correlation metric

The pair correlation metric $S_{\sigma }(i)$ is a tool to analyse the shape and structure of a colony, specifically focusing on how its growth pattern deviates from a circular shape. This metric quantifies the ‘fingering’ or branching patterns that emerge as the colony grows. It is calculated in three steps on a net growth image:

1. We consider all possible pairs of pixels in the colony area and measure the angles between them relative to the centroid $\vec{s}$.
2. These angles are then sorted into $N_{p}$ bins, each representing a small range of angles.
3. We count how many pixel pairs fall into each angle bin.

(2.4) $$\begin{array}{c} S_{\sigma }(i)=\{(\vec{v_{j}}(x,y),\vec{v_{k}}(x,y))|\\ \frac{\pi }{N_{p}}i\leq cos^{-1}\left(\frac{\vec{v_{j}}\cdot \vec{v_{k}}}{|\vec{v_{j}}||\vec{v_{k}}|}\right)<\frac{\pi }{N_{p}}(i+1),\vec{v_{j}},\vec{v_{k}}\in V\text{and }\vec{v_{j}}\neq \vec{v_{k}}\}\\ \text{for i }=0,1,...,N_{p}-1. \end{array}$$

In this work, we chose $N_{p}=180$.

To compare pair correlation metrics across different image sizes, we normalize the pixel pair count in each angular bin by the total number of vector/pixel pairs $|V_{pair}|=\frac{\left(|V|((|V|-1)\right)}{2}$ divided by the number of angular bins $N_{p}$. Given that $|V_{pair}|$ scales with $O(|V{|}^{2})$, computability becomes increasingly challenging for larger images. Therefore, we use a random subset of pixel pairs $V_{sub}$ [15] instead of all possible pairs $V_{pair}$ for larger images. Our tests show that if


$$\begin{array}{rl} & |V|<<|V_{sub}|<<|V_{pair}|\\ & N_{p}<0.001|V_{sub}| \end{array}$$

it is


$$S_{\sigma }(i{)}_{V_{pair}}\approx S_{\sigma }(i{)}_{V_{sub}}.$$

**Figure 5.**
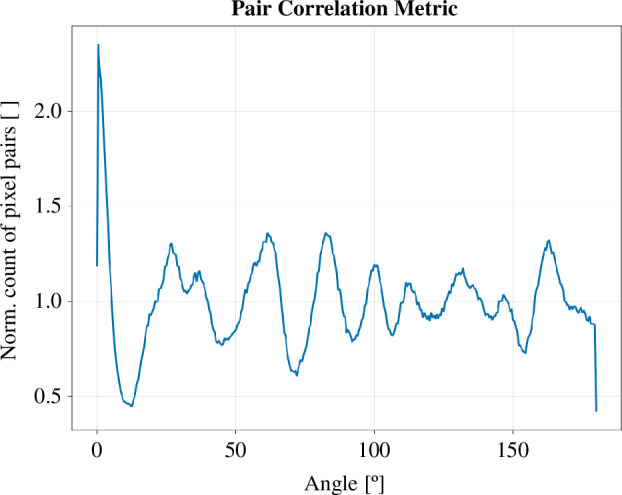
Pair correlation metric for $N_{p}$ = 180, for the net growth area of the colony shown in figure 3.

Figure 5 shows the normalized pair correlation metric for a colony’s growth area. The peaks in this graph represent angles where pixel pairs are more common than average. In a perfectly circular colony, the graph would be flat.

However, in the context of a finger-like geometry, the first and largest peak signifies the accumulation of pixel pairs along the same finger. The distances between subsequent peaks represent the average separation between a finger and its nearest, next-nearest and subsequent neighbour fingers.

To quantify how much a colony deviates from a circle, we use MaxP1 (equation (2.5)). This measures how much higher the largest peak is compared with the graph’s average height. A larger MaxP1 indicates more pronounced finger-like structures


(2.5) $$MaxP1=\max(S_{\sigma })-\bar{S_{\sigma }}.$$

### 2.5. Implementation

Unless otherwise stated, analyses and simulations were performed in Julia [20] and its package Dataframes.jl [27]. All visualizations have been created with the package Makie.jl [28]. The used code base is wrapped into a local Julia package and available on Github.

## 3. Results

### 3.1. *In vitro Trypanosoma* colonies show formation of finger-like structures

We placed droplets of trypanosomes in suspension onto an agarose gel and imaged their evolution over 48 h (figure 1). The colonies show a clear formation of finger-like structures. As a basis for the subsequent quantitative analyses, we performed segmentation and registration of the colony images. We performed four experiments with eight colonies each, resulting in a dataset consisting of 32 colonies that were imaged at three to six different time points. This dataset was used in all following analyses and labelled Exp 1.

### 3.2. *Trypanosoma* colonies exhibit an exponential increase in area

**Figure 6.**
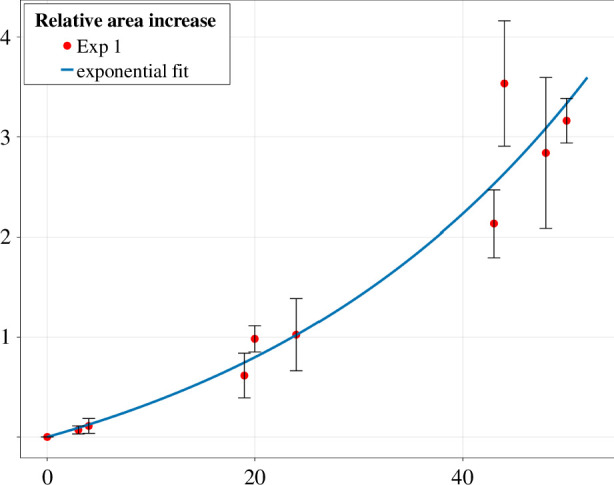
Mean relative area increase over 48 h in 32 colonies from dataset Exp 1 is fitted with an exponential. The error bars represent the standard deviation.


First, we analysed the relative area increase of the *Trypanosoma* colonies over time (figure 6). The measured area was normalized in all colonies to the area of each colony at $A(t=0)=A_{0}$. Given the typical monolayer arrangement of *Trypanosoma* colonies has a consistent density in our experiments, it is reasonable to assume that the increase in colony area A is directly proportional to the increase in cell number. Assuming that nutrient concentration is not a limiting factor within the observed time spans, we presume exponential growth in the number of trypanosomes with growth rate r, hence,

(3.1) $$\begin{array}{r} A(t)=r^{t} \end{array}.$$

The exponential fit results in a doubling time of $\theta =\frac{1}{log_{2}(r)}\approx 23.64\text{ h}$ for the area of the *Trypanosoma* colonies.

### 3.3. Quantitative analyses suggest dynamic changes in the expansion behaviour of *Trypanosoma* colonies

**Figure 7.**
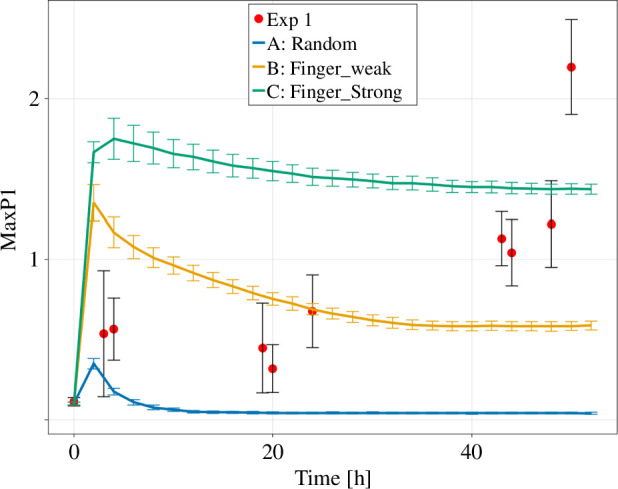
The relative maximum peak height MaxP1 of the pair correlation metric for the three versions of the Eden model and the experimental *in vitro* data. The data points are shown as mean ± standard deviation. The lines are connecting the data points.

We applied pair correlation metric to the experimental as well as artificial data generated by variations of the Eden model and calculated the relative maximum peak height MaxP1 (figure 7).

We find for the artificially created colonies that after an initial transition phase, the value for MaxP1 approaches a constant value. For version A of the Eden model, MaxP1 approaches a value close to zero, as the pixels are very close to a spherical distribution with a uniform probability to find pixels under any angle. In contrast, version B, random growth in combination with directed fingering, displays MaxP1 values ranging between 0.8 and 0.5. Notably, version C, the directed finger growth, maintains the highest MaxP1 values exceeding 1.0 consistently. The initial peak in all three versions can be explained by the limited amount of added pixels in the first time steps, which naturally creates an anisotropy in their distribution.

The *in vitro* colonies show a very different behaviour where MaxP1 exhibits an increase over time, in contrast to the static values observed in *in silico* created colonies. This suggests a dynamic change in the expansion behaviour of real systems. Interestingly, the value of MaxP1 for real colonies, while similar to the hybrid Eden model B for small time points, gradually increases and first approaches and then exceeds the values observed for version C of the Eden model with restricted growth directions.

**Figure 8.**
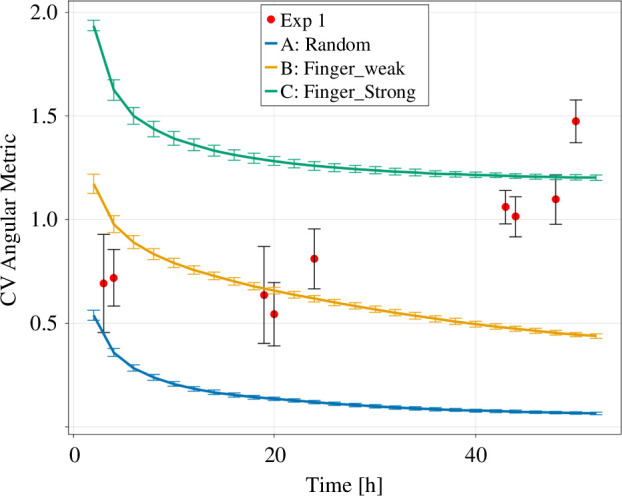
The coefficient of variation (CV of the angular metric for the three versions of the Eden model and the experimental *in vitro* data. The data points are shown as mean ± standard deviation. The lines are connecting the data points.

To test these results, we next performed analyses based on the angular metric. This metric describes how the colony’s edge varies when moving along its circumference. The metric can be thought of as a periodic signal, such that a flat signal represents circular growth and a fluctuating signal represents finger-like growth. To quantify these fluctuations, we used the coefficient of variation (CV), a standard measure in signal analysis. The CV is calculated by dividing the standard deviation by the mean (CV=σ/μ) [29]. In the context of the trypanosome colonies, the CV becomes a useful tool for measuring how ‘finger-like’ a colony is (figure 8).

**Figure 9.**
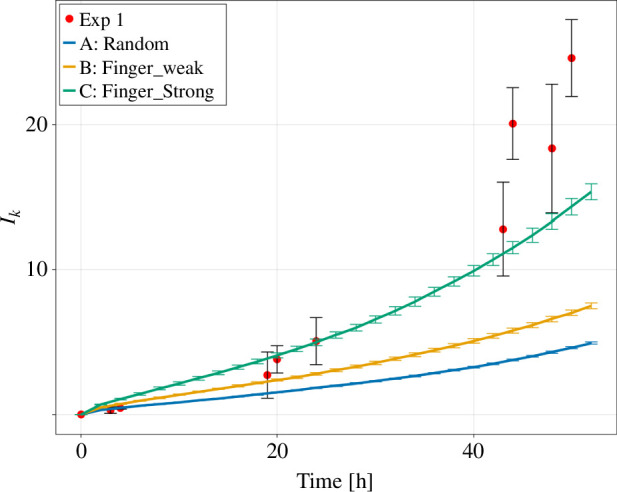
The normalized average amplitude $I_{k}$ of the Fourier-transformed angular metric for the three versions of the Eden model and the experimental *in vitro* data. The data points are shown as mean ± standard deviation. The lines are connecting the data points.

We calculated the CV for both the *in vitro* colonies and the artificially generated data (figure 8). Similar to the MaxP1, the CV shows an asymptotic decrease for all three *versions* of artificially created colonies (figure 8). This is again due to the random growth together with the low number of added pixels for smaller time steps, which initially creates a larger anisotropy in the distribution of growth sites.

Version A of the Eden model approaches zero for bigger time steps, which is expected as the signal becomes more and more isotropic for larger pixel numbers. Version B assumes larger values between 1.2 and 0.4 as the partially restricted growth prevents the signal from becoming constant. Version C assumes the highest values between 1.9 and 1.2, as the pure restricted growth has the largest fluctuations in the angular metric.

The real colonies show a different behaviour, where the CV initially assumes similar values as for version B but increases over time and assumes values larger than for version C.

Considering the angular metric as the periodic finger signal suggests a Fourier transformation for further analysis. The Fourier transformation produces the spectrum of frequencies present in our finger signal, each weighted by its amplitude. We normalized the Fourier-transformed signal by the initial area of each colony ($A_{0}$), resulting in the following equation:


(3.2) $$|{\hat{f}}_{k}|=\frac{1}{A_{0}}\left|\sum_{n=0}^{N-1}S_{\theta }(n)e^{\frac{-i2\pi kn}{N}}\right|,$$

where $|{\hat{f}}_{k}|$ represents the normalized amplitude of each frequency component (spectral density).

As the first component of ${\hat{f}}_{k}(S_{\theta })$ corresponds to the offset of the signal from zero, which is proportional to the net growth area of the colony, it is neglected in all further analyses. In line with that, the mean of all amplitudes $I_{k}$ for k>1 is calculated as


(3.3) $$\begin{array}{ccc} & I_{k}=\frac{1}{N-2}\sum_{k=2}^{N-1}|{\hat{f}}_{k}|. & \end{array}$$

$I_{k}$ can be interpreted as a measure for the amount of periodic fluctuations of the surface of a colony.

$I_{k}$ only increases slightly over time in the Eden model in version A, which grows almost in a constant uniform circular fashion (figure 9). Version B shows a much stronger increase over time and version C the strongest as it exclusively grows in a non-circular way. The *in vitro* colonies show again a dynamic transition between the artificial cases. Initially, their $I_{k}$ starts near 0 as versions A and B; after about 20 h, $I_{k}$ is between B and C and after about 48 h, $I_{k}$ exceeds version C.

A second possible interpretation of the angular metric is as the grown surface of the colony projected into one spatial dimension. Hence, measures to quantify surfaces can be applied. We used a common measure of surface roughness, the root-mean-squared deviation of the height h(x), often called the global interface width W [30]. To make this surface roughness independent of system size, we normalized the height measurements by the size of the area of each individual colony at $A(t=0)=A_{0}$ and obtained


(3.4) $$\begin{array}{r} h_{n}(i)=\frac{S_{\theta }(i)}{A_{0}} \end{array}$$

and


(3.5) W(N,t)=⟨(hn(i,t)−hn¯)2⟩.

The overbar denotes averaging over all i in a single system of size N and the ⟨-⟩ denotes averaging over an ensemble of n systems.

**Figure 10.**
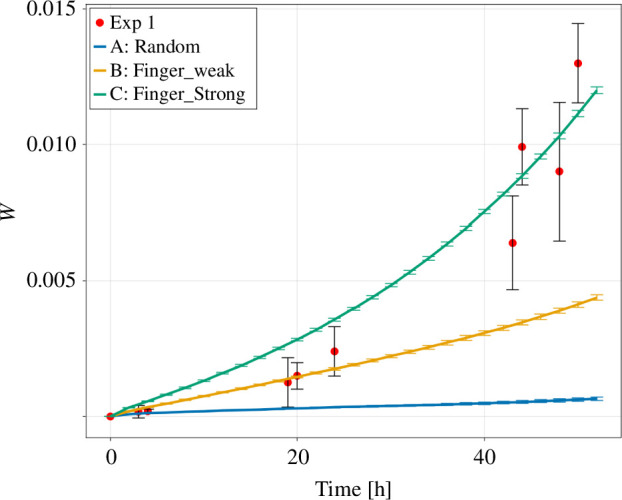
The surface roughness for the three versions of the Eden model and the experimental *in vitro* data. The data points are shown as mean ± standard deviation. The lines are connecting the data points.

The Eden model in version A shows only a small increase in W in the observed time frame. The versions B and C are far away from this regime and their roughness shows a stronger increase over time. This increase in roughness is stronger in version C. The *in vitro* colonies show again a dynamic switch. Hence, their roughness is initially close to 0, then after about 20 h most similar to version B and after about 48 h most similar to version C of the Eden model (figure 10).

In conclusion, all four measurements show a clear distinction for the three different versions of the Eden model. Comparing these results to the measurements for the *in vitro* data suggests that the *Trypanosoma* colonies first grow randomly and then restricted growth within specific directions becomes increasingly dominant.

### 3.4. Growth phases are robust with respect to the number of cells and inhibition

**Figure 11.**
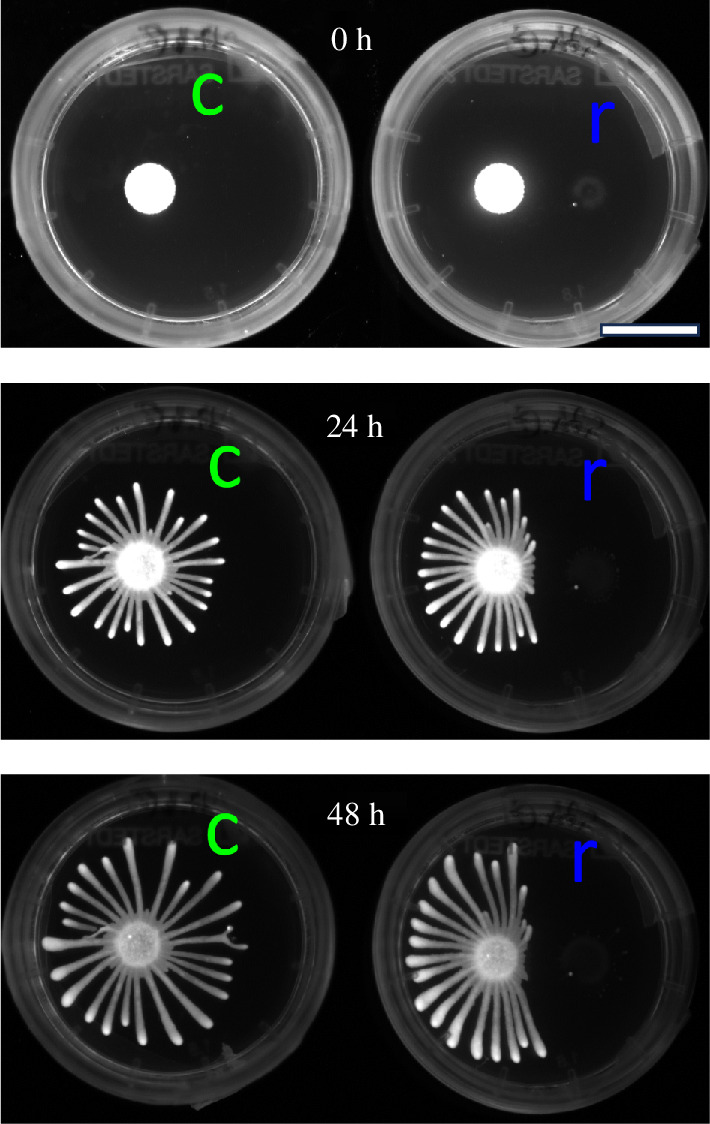
Time-series fluorescence images of two *Trypanosoma* colonies from a single assay (Exp 2). The right colony (r) is hindered by a non-fluorescent SoMo negative colony, while the left control colony (c) expands unimpeded. A contrast-enhanced image highlighting the SoMo negative colony is provided in figure 20 (appendix A). Scale bar: 20 mm.

To assess the robustness of different growth phases, an additional assay was conducted involving six colonies. Two colonies were subjected to repulsion by a SoMo negative trypanosome colony (Exp 2: repelled), while four served as control colonies without repulsive signal (Exp 2: control). In addition, for all six colonies, the initial cell number was larger than in the previous experiments. This led to accelerated expansion and the formation of a larger number of fingers (approx. 30) (figure 11).

These colonies exhibited slightly different area growth dynamics due to their initial non-monolayer configuration. We employed a modified exponential growth model to fit the area data and calculated the relative area increase over time (see appendix B for further details).

**Figure 12.**
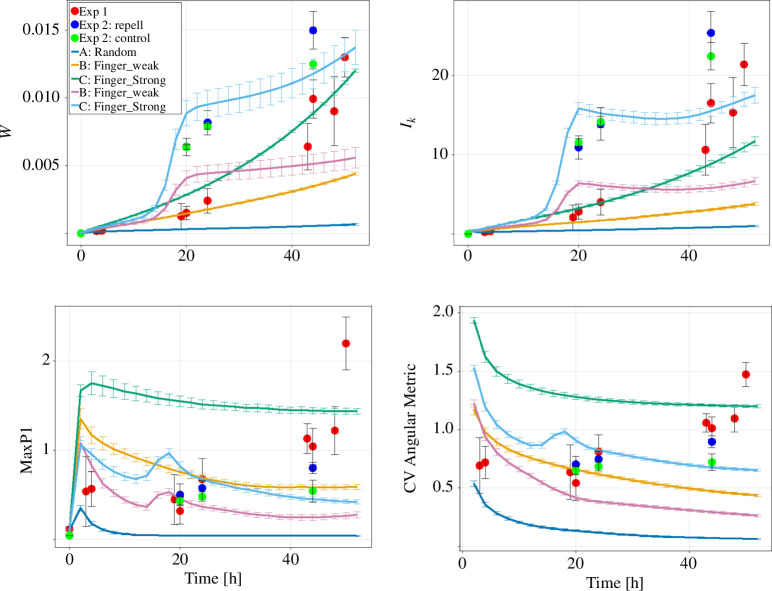
$W,I_{k},MaxP1,CV$ are shown for the previous simulated and *in vitro* colonies (Exp 1) together with control, repelled (Exp 2) and simulated colonies with 30 fingers and accelerated expansion. The data points for repel and control colonies for $W,I_{k},MaxP1$ at *t* = 0 are overlapping at zero.

We simulated colonies with these modified growth dynamics and 30 fingers, then applied our established metrics to the additional data. We found that W and $I_{k}$ exhibited an increase for colonies with more fingers, while MaxP1 and CV decreased (figure 12). This trend held true for both simulated and real colonies. The peaks in these measurements just before 20 h relate to the altered growth dynamics of the colonies with more cells (see appendix B for further details). We further find that control colonies from Exp 2 display the same transition behaviour from circular to finger-like growth as the colonies from Exp 1, proving the robustness of our methods. A slight difference between control and repelled colonies was observed, albeit inconclusive. Hence, an alternative quantification of this geometric change is required.

### 3.5. Quantification of deflection

**Figure 13.**
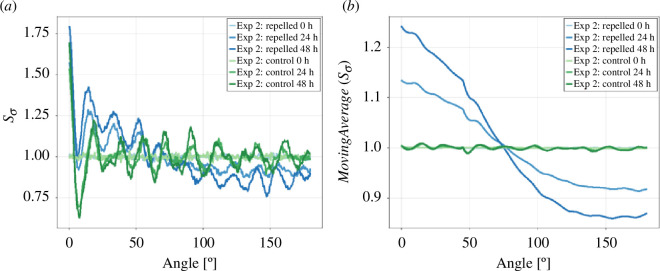
*(a*) The mean pair correlation metric $S_{\sigma }$ of repelled and control colonies of Exp 2. (*b*) The moving average of the mean pair correlation metric $S_{\sigma }$ for a window of 90° (MA) shows a distinct difference between repelled and controlled colonies.

To quantify the deflection, we examined the pair correlation metric $S_{\sigma }$, which assesses the anisotropy in the pixel distribution (figure 13*a*). We find a distinct differentiation between control and repelled colonies, characterized by an upward shift for small angles and a downward shift for larger angles. To pinpoint this further, we smoothed $S_{\sigma }$ with a moving average (MA) of $S_{\sigma }$ for a window of 90° (figure 13*b*).

We propose the anisotropy index $A_{I}$, quantifying the relative relationship between the maximum and minimum values of the smoothed pair correlation metric $S_{\sigma }$ as


(3.6) $$A_{I}=\frac{max(MA(S_{\sigma }))-min(MA(S_{\sigma }))}{max(MA(S_{\sigma }))+min(MA(S_{\sigma }))}.$$

We computed the mean $A_{I}$ for all repelled and control colonies in the additional assay, alongside the four previous assays and the simulated colonies.

**Figure 14.**
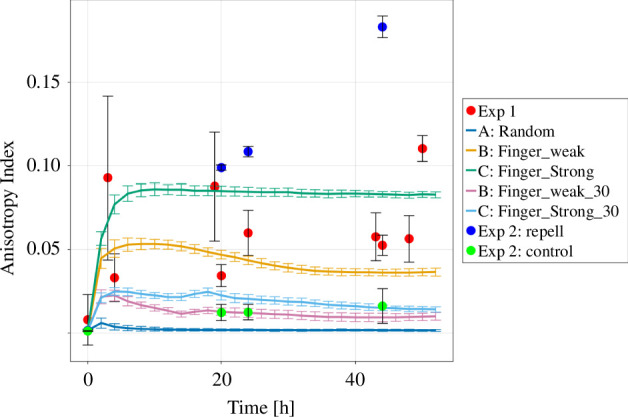
The anisotropy index for the three versions of the Eden model, with 15 fingers and 30 fingers, respectively, as well as the experimental *in vitro* data. The data points are shown as mean ± standard deviation. The data points for repel and control colonies at *t* = 0 are overlapping at zero. The lines are connecting the data points.

We note that the anisotropy index $A_{I}$ tends to be larger for colonies with fewer fingers (both real and simulated) (figure 14), which is reasonable considering that fewer fingers indicate a less isotropic distribution of pixels. The real colonies exhibit values ranging between 0.05 and 0.1, akin to the simulated colonies of models B and C. For the last time point, the real colonies have a surprisingly large anisotropy value. This result matches with visual inspection of the corresponding images as the fingers are quite different in size in the corresponding dataset. To investigate whether this is an artefact or a relevant behaviour could be investigated in future studies. Conversely, for colonies with more fingers, $A_{I}$ is smaller, with control colonies displaying similar values to the simulated ones with 30 fingers, hovering around 0.01. Notably, repelled colonies exhibit the highest values, surpassing 0.1 after 24 h and reaching 0.16 after 48 h, clearly distinguishing them from the control colonies. A short summary of used metrics can be seen in InfoBox 1.

InfoBox 1. **Short summary of all used metrics.**

Summary

MaxP1:

— Derived from the pair correlation metric
— Measures the prominence of finger-like structures

*CV* (coefficient of variation):

— Derived from the angular metric
— Higher values indicate more pronounced finger-like structures

*I*_*k*_ (mean Fourier amplitude):

— Obtained from Fourier transformation of the angular metric
— Quantifies periodic surface fluctuations of the colony

*W* (global interface width):

— Measures surface roughness of the colony
— Increases as growth becomes more irregular or finger-like

*A_I_* (anisotropy Index):

— Based on the smoothed pair correlation metric
— Measures directional bias in colony growth

general trends:

— W and *I*_*k*_ and increase for colonies with more fingers, while MaxP1 and CV decrease
— *A*_*I*_ is larger for colonies with fewer fingers

## 4. Discussion

We established quantification methods for *Trypanosoma* social motility assays. Our measurements allow objective evaluation of colony and finger growth dynamics. We find that the trypanosome colonies exhibit a dynamic growth process that transitions from initial pure circular expansion to exclusive ‘finger’ expansion. These growth phases are independent of the initial number of cells and anisotropies in colony expansion.

Fluorescent images were processed in a multi-stage image analysis pipeline to enable the calculation of the angular metric and pair correlation metric, two one-dimensional metrics introduced by Binder [15]. We measured the area increase over time to simulate three different randomly growing colonies based on the phenomenological Eden model growth model [16].

The area doubling time derived from our fitting exceeds the population doubling time reported in the literature [9,13] but is within the margin of error of the reported doubling times of the mono layer assay we used [14]. This means that cell growth is the primary driver of colony expansion, and the area increase could be used as a proxy for cell growth.

The angular and pair correlation metrics were further processed by calculating five quantities ($MaxP1,CV,W,I_{k},A_{I}$) that map the growth processes to scalar values. Calculating these measures for the different Eden models shows that these quantities differentiate between different growth modes. They assume the lowest values for random growth and increase with increasing contribution of finger growth.

For the simulated colonies, the relative maximum peak of the pair correlation metric MaxP1 and the coefficient of variation of the angular metric CV decrease over time. This can be explained by the constant growth rules in the simulated colonies, which cause both these quantities to asymptotically approach a constant value for t→∞. In the real colonies, however, both these quantities increase over time, which is caused by the changing expansion behaviour from spherical to finger-like. Both MaxP1 and CV are sensitive to a higher number of fingers and show overall lower values due to the less pronounced single fingers compared with the average signal. Inhibition does not have a significant impact on their values.

The surface roughness W and normalized Fourier coefficients $I_{k}$, even though measuring quite different quantities of the system, showed very similar qualitative behaviour. Both increased over time for the simulated colonies. The transition from random circular to finger-like growth behaviour in real colonies can again be mapped here. Contrary to MaxP1 and CV, both W and $I_{k}$ increase with increasing numbers of fingers. Inhibition also does not have a significant impact on their values.

Since the previous four quantities failed to distinguish between normal and inhibited colonies, a fifth quantity was developed: the anisotropy index $A_{I}$. This metric assigns a numerical value to the anisotropy of a colony. While it excels at differentiating between inhibited and normal colonies, it may also prove useful for comparing the uniformity of growth between colonies. This allows for the accounting of anisotropies in the experimental set-up, variations in cell behaviour and other factors.

Taken together, our methods demonstrate the possibility for an objective description of the behaviour typically arising in trypanosome social motility assays. The combination with the Eden model allows for categorizing the behaviour types observed. This approach is not limited to trypanosomes but can be directly applied to other colony-forming cells like yeast [31,32] or bacteria [33–35]. Especially the very similar swarming patterns of *Pseudomonas* [5] present a promising application for our metrics to quantitatively validate the available modelling approaches for that system.

Application of our methods to *in vitro* trypanosome colonies reveals that simple growth dynamics as represented by the Eden model are not sufficient to accurately capture social motility dynamics. This is not too surprising as social motility is most likely a consequence of cell movement, hydrodynamics, chemotaxis and cell growth. Therefore, a model that accurately represents SoMo must incorporate more of these effects. However, in combination with data from single cells [14], our colony-scale quantification builds a solid basis for developing and quantitatively validating such models as has recently been done for yeast colonies [36]. Hence, we provide a step towards data-driven mechanistic modelling of social motility *in vitro*.

## Figures and Tables

**Figure 15.**
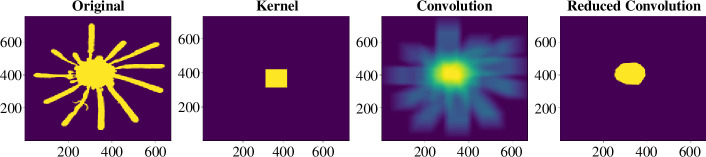
Convolution of a colony binary image with a rectangular kernel creates an intensity image, which again is binarized.

**Figure 16.**
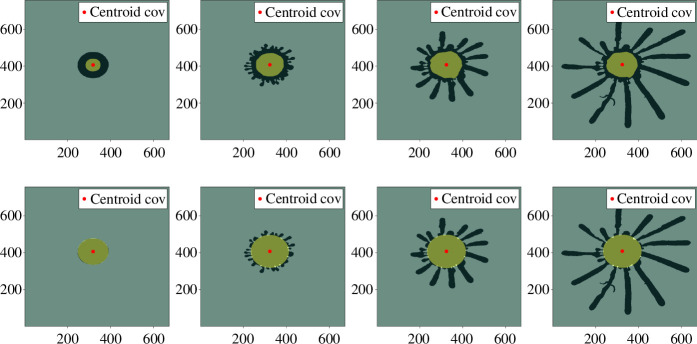
Time series of a colony shown together with the reduced colony and its centroid produced by convolution (top) and together with the fitted circle from the centroid of the reduced image (bottom).

**Figure 17.**
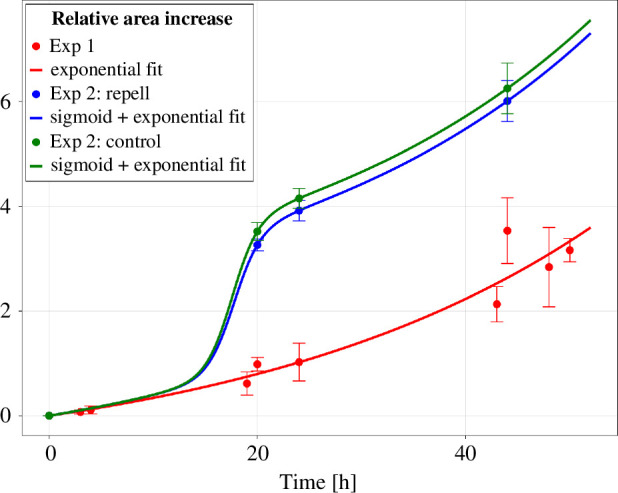
Mean relative area increase over 48 h for the three different datasets fitted with exponential (and sigmoid functions). The error bars represent the standard deviation.

**Figure 18.**
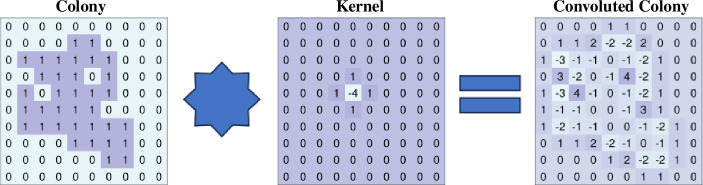
The convolution of a Laplace kernel with the colony lattice extracts all perimeter and surface sites. Perimeter sites assume positive values corresponding to their adjacent surface sites. Surface sites assume negative values corresponding to their adjacent perimeter sites.

**Figure 19.**
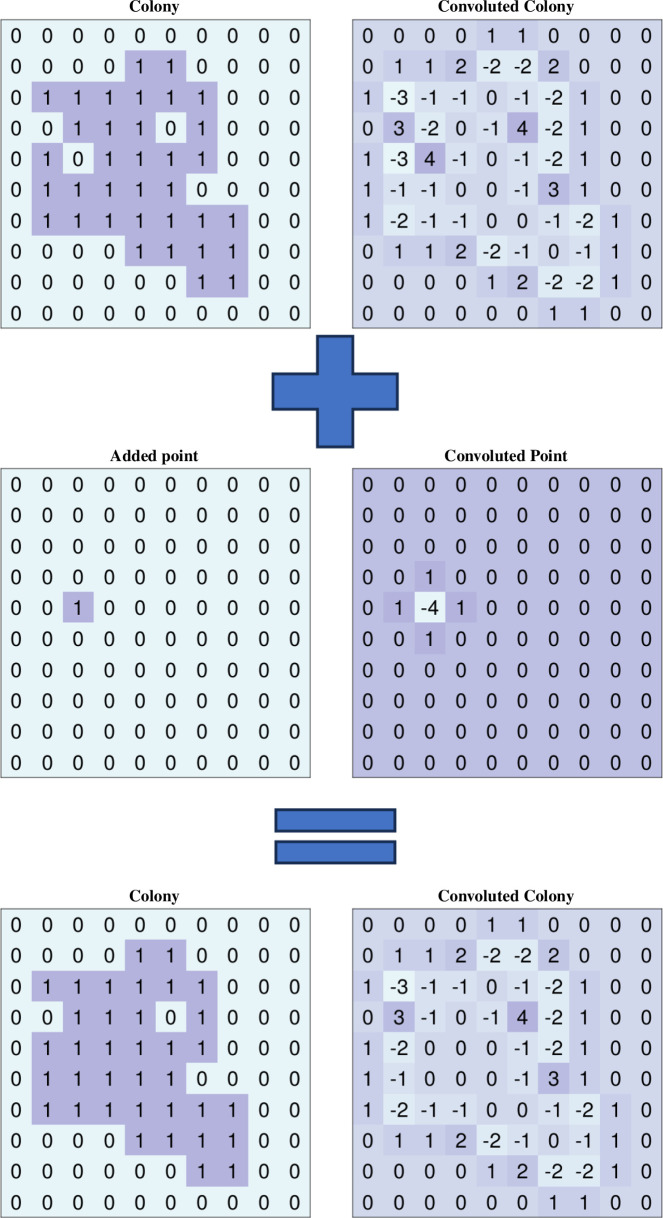
The colony and auxiliary lattice get updated simultaneously. A filled site in the colony corresponds to an added Laplace kernel at the same position in the auxiliary lattice.

**Figure 20.**
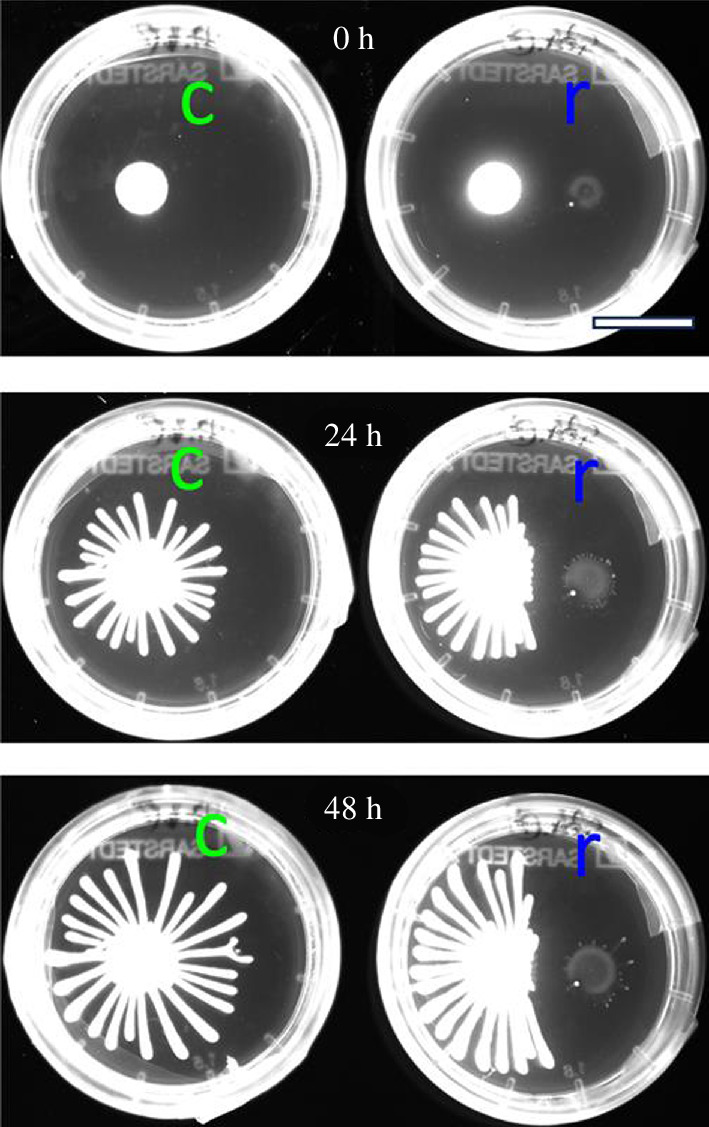
Contrast-enhanced version of figure 11 showing the interaction between fluorescent and non-fluorescent *Trypanosoma* colonies. The SoMo negative, non-fluorescent colony is now visible as the contrast is enhanced, demonstrating its hindrance of the fluorescent colony’s expansion. The small white circle in the SoMo negative colony is a small crater produced by a collapsed bubble on the gel surface where trypanosomes collect and swim in the fluid. This does not affect the general behaviour. Scale bar: 20 mm.

## Data Availability

The Fiji scripts for data processing together with the Julia package for analysis are available in their newest version via GitHub [37]. The version of the software that was used to create this publication together with the used data is available in this Zenodo repository [38].

## Declaration of AI use

Github Copilot was used for the automatic creation of code documentation of Colonyimages.jl and to increase coding efficiency during development. ChatGPT and Claude were used to improve the readability and language in parts of the manuscript.

## Appendix A. Detailed description of the image analysis pipeline

The fluorescence images were preprocessed with a Gaussian filter (σ=1.5). This step helps prevent false detections caused by uneven illumination, particularly in thinner and less bright regions of finger structures. The segmentation was done by the machine learning Fiji [17] plugin Trainable Weka Segmentation [18]. One of the images of a colony at *t* = 48 h was used for initial training. Areas of the background and colony have been manually selected to serve as the basis for the training of a classifier. After the application of the classifier on multiple colonies and time points, the selected areas were manually adapted to optimize further training.

Subsequently, the Fill Holes function provided by MorphoLibJ [39] was applied to binary images to eliminate falsely classified holes fully contained within the colony. Additionally, artefacts such as the bright edge of the agarose gel plate, incorrectly identified as part of the colony, were manually removed.

In the next stage, binary images of the same colony were stacked and aligned (figure 1 in the main text) using the Rigid Body transformation type of the Fiji plugin StackReg [19]. Subsequently, the first image (*t* = 0 h) was subtracted from later images to isolate the net grown area for further analysis.

Later analysis showed that the pair correlation metric $S_{\sigma }$ for small time steps (*t* < 10 h) is very sensitive to small shifts/errors in the alignment process causing anisotropies in the net growth images $I_{net}$, even though the colony expands perfectly circular in that time frame. If this was confirmed manually, an alternative technique was used to isolate the net grown area from the original colony structure relying solely on the information contained in a single image. This approach used the following assumptions:

— All colonies start out with an almost perfect spherical shape.
— Expansion is majorly in the form of asymmetrical protrusions which rapidly evolve into distinct finger-like shapes.

The first step is to calculate the convolution of the binary colony image with a rectangular kernel roughly 40% of the area of the original colony (figure 15). This convolution yields an intensity image that is binarized with a threshold of 0.8 where all pixels below that threshold are set to zero and all above to one (figure 16*a*).

Then, the centroid $\vec{s}$ equation (2.2) of the reduced convolution image is calculated and used to fit a circle into the original binary colony (figure 16*b*). This circle should closely approximate the colony’s initial spherical state at t=0. The circle is gradually built up from the centroid of the colony. If less than 50% of the grid points on the outermost annulus of the circle are occupied, the fitting is stopped.

This method offers an alternative approach to differentiate between the spherical ‘core’ region and the newly developed finger-like regions. Compared with the stacking method, this approach provides less precise but more robust results. We have calculated all our results for both methods, and they did not exhibit significant differences, except for the described issues encountered during small time points in the calculation of the pair correlation metric $S_{\sigma }$. Therefore, we employed the Fiji-based stacking method for all our results, where this issue did not arise, and the convolution method for the few datasets where there were alignment problems for small time steps. We meticulously reviewed all the results to ensure their validity.

## Appendix B. Area fitting of colonies with 30 fingers

A sole exponential fit to model the relative area increase over time for the additional assays featuring colonies of increased cell numbers yielded unsatisfactory results. The elevated cell density in these colonies prompted a rapid initial area increase as the colonies probably thinned towards their preferred monolayer arrangement. Simultaneously, exponential growth due to cell division contributed to overall area expansion. To effectively capture this dual behaviour, a composite approach combining a sigmoid-like function to represent thinning and exponential growth to model cell division was employed. This combined model demonstrated good agreement with the observed data.

The logistic function was chosen as the sigmoidal component due to its simplicity and ease of use, although this selection was somewhat arbitrary, given the limited data points available to precisely characterize thinning-induced area increase. However, this choice is not anticipated to significantly impact subsequent analyses, which predominantly focus on time points post-thinning (figure 17).

The composite fitting function is expressed as

(B 1)
(3)A(t)=rt+L1+e−p(t−t0).

The exponential segment of the fitting function yielded doubling times of approximately $\theta =\frac{1}{log2(r)}\approx 20.96\text{ hours}$ for the repelled colonies and θ=1/r≈20.91 hours for the control *Trypanosoma* colonies.

## Appendix C. Implementation of the Eden model

The Eden model was implemented in the programming language Julia [20], enabling growth simulations of 30 colonies with up to $10^{6}$ added sites in under 10 min. The chosen variant of the model tracks all perimeter sites, defined as empty sites with at least one surface site as a neighbour. A random selection process designates one of these sites for population, followed by an update of the perimeter sites list. Algorithmically, this operation can be executed through the convolution of a Laplacian kernel with the lattice. The result is an auxiliary lattice with the same size as the original lattice. All perimeter sites in the auxiliary lattice assume positive values corresponding to their adjacent surface sites, surface sites assume the negative count of adjacent perimeter sites and all other sites assume a zero value (figure 18).

The auxiliary lattice is subsequently filtered to extract only those sites with positive values, thereby isolating the perimeter sites. Upon filling one of these sites (e.g. setting its value to one in the main lattice), a single Laplacian kernel is added to the auxiliary lattice centred around the site, triggering automatic updates for all neighbouring sites accordingly (figure 19). This results in a run time O(M) that is linear with respect to the size M of the used lattice.

## Appendix D. Time-series fluoroescence image with enhanced local contrast

See figure 20.
