# Correction: ‘Quantification of *Trypanosoma brucei* social motility indicates different colony growth phases’ (2024), by Kuhn *et al*.

**DOI:** 10.1098/rsif.2025.0568

**Published:** 2025-08-20

**Authors:** Andreas Kuhn, Timothy Krüger, Magdalena Schüttler, Markus Engstler, Sabine Christine Fischer

**Affiliations:** ^1^ Center for Computational and Theoretical Biology, Julius-Maximilians-Universität Würzburg Fakultät für Biologie, Wurzburg, Bayern 97074, Germany; ^2^ Biozentrum, Julius-Maximilians-Universität Würzburg Fakultät für Biologie, Wurzburg, Bayern 97074, Germany

*J. R. Soc. Interface* 21: 20240469. (Published online 18 December 2024). (https://doi.org/10.1098/rsif.2024.0469)

We accidentally used the formula for doubling time applicable to linear growth instead of the correct exponential growth formula. When corrected, this leads to the following amendments.

In §3.2, the last sentence is changed to:

The exponential fit results in a doubling time of $\theta =\frac{1}{log_{2}(r)}\approx 23.64\text{ h}$ for the area of the *Trypanosoma* colonies.

In the discussion, the paragraph discussing the doubling time is changed to:

The area doubling time derived from our fitting exceeds the population doubling time reported in the literature [1,2] but is within the margin of error of the reported doubling times of the monolayer assay we used [3]. This indicates that cell growth is the primary driver of colony expansion, and the area increase could be used as a proxy for cell growth.

In appendix B, the last sentence is changed to:

The exponential segment of the fitting function yielded doubling times of approximately $\theta =\frac{1}{log2(r)}\approx 20.96\text{ h}$ for the repelled colonies and θ=1/r≈20.91 hours for the control *Trypanosoma* colonies.

All other results and the conclusions drawn are unaffected.
